# Chemical Fingerprint Analysis and Quantitative Analysis of *Rosa rugosa* by UPLC-DAD

**DOI:** 10.3390/molecules21121754

**Published:** 2016-12-21

**Authors:** Sanawar Mansur, Rahima Abdulla, Amatjan Ayupbec, Haji Akbar Aisa

**Affiliations:** 1 Key Laboratory of Xinjiang Indigenous Medicinal Plants Resource Utilization, Xinjiang Technical Institute of Physics and Chemistry, Chinese Academy of Sciences, Urumqi 830011, China; sanam0405@163.com (S.M.); rahima@ms.xjb.ac.cn (R.A.); amatjanayupbig@hotmail.com (A.A.); 2 Key Laboratory of Plant Resources and Chemistry in Arid Regions, Xinjiang Technical Institute of Physics and Chemistry, Chinese Academy of Sciences, Urumqi 830011, China; 3 University of Chinese Academy of Sciences, Beijing 100049, China

**Keywords:** *Rosa rugosa*, UPLC-DAD, quality control, fingerprint

## Abstract

A method based on ultra performance liquid chromatography with a diode array detector (UPLC-DAD) was developed for quantitative analysis of five active compounds and chemical fingerprint analysis of *Rosa rugosa*. Ten batches of *R. rugosa* collected from different plantations in the Xinjiang region of China were used to establish the fingerprint. The feasibility and advantages of the used UPLC fingerprint were verified for its similarity evaluation by systematically comparing chromatograms with professional analytical software recommended by State Food and Drug Administration (SFDA) of China. In quantitative analysis, the five compounds showed good regression (R^2^ = 0.9995) within the test ranges, and the recovery of the method was in the range of 94.2%–103.8%. The similarities of liquid chromatography fingerprints of 10 batches of *R. rugosa* were more than 0.981. The developed UPLC fingerprint method is simple, reliable, and validated for the quality control and identification of *R. rugosa*. Additionally, simultaneous quantification of five major bioactive ingredients in the *R. rugosa* samples was conducted to interpret the consistency of the quality test. The results indicated that the UPLC fingerprint, as a characteristic distinguishing method combining similarity evaluation and quantification analysis, can be successfully used to assess the quality and to identify the authenticity of *R. rugosa*.

## 1. Introduction

Traditional Chinese medicine (TCM) has recently become an attractive subject for many scientists and drug producers. Many forms of TCM, across history and cultures, have been used for medicinal purposes as alternative therapies based on plants in order to avoid drug adverse effects, and many articles have been written in recent years. When TCM is used, particular attention must also be paid to the standardization process. Additionally, the Food and Drug Administration (FDA) specifies certain labeling requirements for foods, supplements, and drugs, and the European Union requires that standardized herbal substances be reported as having constituents with known therapeutic activity.

*Rosa rugosa* Thunb. (Family Rosaceae), which is distributed throughout the temperate regions of Eastern Asia including China, Japan, and Korea, is conventionally used as medicinal plants in TCM. In Asia, it is a form of traditional herbal medicine for treating stomach ache, diarrhea, menoxenia, pain, and chronic inflammatory diseases [1]. Phytochemical studies conducted so far have shown the isolation of tannins, flavonoids, terpenoids, triterpenoids, steroids, tocopheral, and carotene [2]. Tannins and flavonoids are of special interest because of their activities, such as antioxidative, antidiabetes, and antinflammatory activities associated with global diseases, including diabetes mellitus, pain, and chronic inflammatory diseases [3].

The qualitative and quantitative analysis of major components and the analysis of chemical fingerprints have been introduced and accepted by the State Food and Drug Administration (SFDA) of China (2000). Different cultivation areas and climatic conditions may vary the chemical constituents of *R. rugosa* significantly. Since application is steadily increasing, the development of a suitable quality control method is urgently required. The official Chinese pharmacopoeia (China Pharmacopoeia Committee 2010) does not include a quality evaluation of *R. rugosa*. 

Among the chromatographic fingerprinting applied to the authentication and qualitative evaluation of botanical products over the past decade, high performance liquid chromatography (HPLC) fingerprinting emerges as the most widely used method because of its convenience and efficiency [4,5,6,7,8,9]. However, the acquisition of a fingerprint and quantitative analysis by these methods has been a tedious operation, as it generally requires about one or more hours for a single run. In recent years, UPLC has emerged as a viable technique for quantitative and chemical fingerprint analysis of natural products, and some reports have appeared in the literatures on its applications in the fingerprinting and quantitative analysis of Chinese herbal medicines [10]. The results obtained in these references demonstrated that UPLC is indeed a very powerful tool in chromatographic fingerprinting applications and quantitative analysis of the components in these herbal medicines.

To the best of our knowledge, there have not been any reports regarding the fingerprint analysis of *R. rugosa*. The objective of this study was to establish an effective UPLC fingerprint method for the identification and quality evaluation of *R. rugosa*. The chromatograms of the extracted samples from different *R. rugosa* plantations of Xinjiang were compared visually and analyzed via similarity evaluation. Moreover, 23 components in 10 batches of *R. rugosa* were simultaneously quantitated via the UPLC method.

## 2. Results and Discussion

### 2.1. Optimization of Analysis Conditions

**Figure 1:**
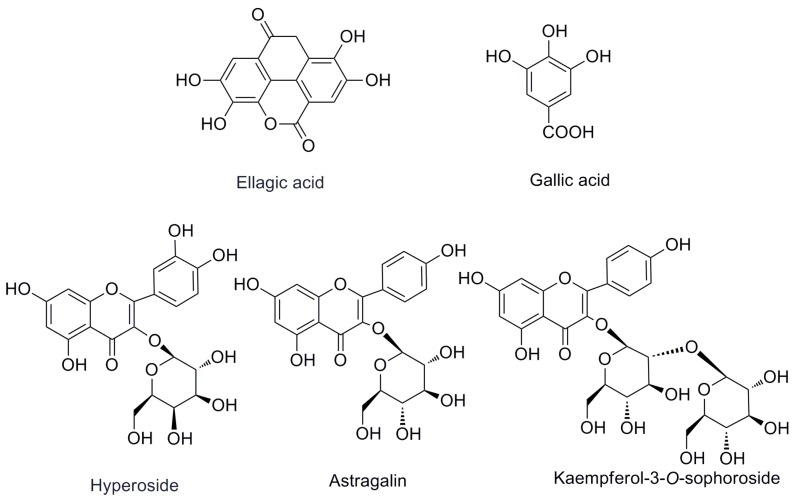
The chemical structures of the investigated compounds.

**Figure 2:**
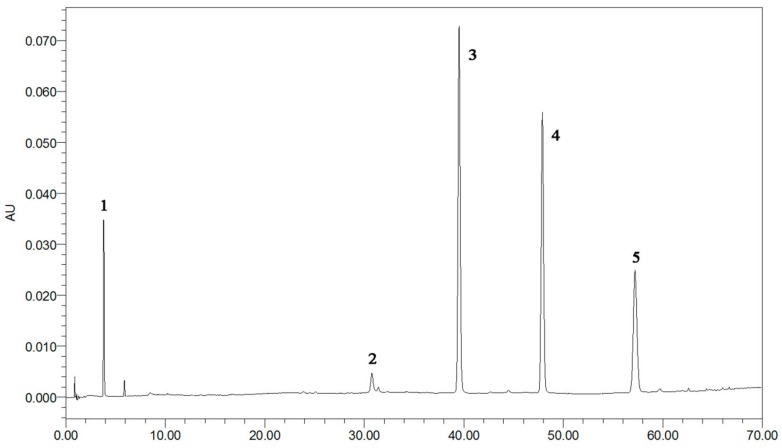
The typical UPLC chromatographic profile of five standards peaks: **1**: GA (3.8 3min); **2**: EA (30.7 min); **3**: kaempferol-3-*O*-sophoroside (38.9 min); **4**: hyperoside (48.9 min); **5**: astragalin (57.2 min).

The first step in the quantitation of the five standards in *R. rugosa* (structures shown in Figure 1 and UPLC chromatographic peaks in Figure 2) is the full extraction from materials [11,12]. The preliminary tests made in this study indicated that the different extraction methods (ultrasonication, reflux, and Soxhlet) have a great influence on the content of the five standards. The contents of the five compounds are higher when subjected to the ultrasonic extraction standards method compared with those subjected to other methods. In order to optimize ultrasonic extraction conditions, different extraction solvents such as methanol, ethanol, and different combinations of water and ethanol were used with ethanol concentrations of 20%, 40%, 60%, 80%, and 95%, *v*/*v*. Extraction time was varied in each case: 30, 40, 50, or 60 min. By comparing the total weight of exact and areas of the characteristic peaks in each chromatogram of different factors, the optimal condition for extraction was selected as below for each sample: 5.0 g of dry powder was extracted with 100 mL of 60% (*v*/*v*) aqueous ethanol in an ultrasonic bath for 60 min.

### 2.2. Optimization of UPLC Chromatographic Conditions

In order to obtain the most useful chemical information and best separation in the fingerprint chromatograms of *R. rugosa*, those conditions including the column, the mobile phase compositions, the gradient elution procedure, and the detection wavelength were optimized. Two kinds of reverse-phase columns, BEH Shield C18 column (100 mm × 2.1 mm, 1.8 µm) and BEH C18 column (100 mm × 2.1 mm, 1.8 µm), were investigated, and the BEH Shield C18 column was found to be more suitable due to its better separation and shapes of peaks.

In order to enhance the resolution and to restrain the ionization of target compounds, formic acid was added to the binary mixture of acetonitrile–water. To acquire better selectivity and higher efficiency, different concentrations of formic acid (0.05%, 0.1%, and 0.2%) in the acetonitrile phase were also investigated. The resulted optimized mobile phase consisting of acetonitrile with 0.1% formic acid (pH 2.98, *v*/*v*), and water with 0.1% formic acid solution (pH 2.67, *v*/*v*) was chosen for the determination of *R. rugosa* with a large number of peaks on the chromatogram achieved within 71 min. More detectable peaks could be obtained, and the baseline was well improved at around 260 nm; therefore, better results for the five target compounds in *R. rugosa* and reference standards could be obtained. Hence, characteristic chromatographic patterns were obtained using 260 nm as the detection wavelength. The optimal UPLC condition used in this study is shown in the UPLC Condition section.

### 2.3. Method Validation of Quantitative Analysis

The method of quantitative analysis was validated in terms of linearity, the limit of detection (LOD), and the recovery test. Linear regression analysis for each compound was performed by plotting the peak area (*y*) against the concentrations (*x*, mg/mL) of the mixed standard solution, which was expressed as follows: *y* = 1.79 × 10^8^*x* − 4.14 × 10^4^, R^2^ = 0.9999 (for GA, the linear range is 0.02–0.1 mg/mL); *y* = 4.58 × 10^8^*x* − 2.03 × 10^5^, R^2^ = 0.9999 (for EA, the linear range is 0.02–0.2 mg/mL); *y* = 7.12 × 10^5^*x* − 2.28 × 10^2^, R^2^ = 0.9995 (for hyperoside, the linear range is 0.05–0.3 mg/mL); *y* = 1.84 × 10^7^*x* − 1.14 × 10^3^, R^2^ = 0.9999 (for astragalin, the linear range is 0.02–0.1 mg/mL); *y* = 1.28 × 10^7^*x* − 9.20 × 10^3^, R^2^ = 0.9999 (for kaempferol-3-*O*-sophoroside, the linear range is 0.02–0.1 mg/mL).

The LOD and LOQ under the present UPLC method were determined at signal–noise ratios (S/N) of 3 and 10, respectively. Standard solution containing five reference compounds was diluted to a series of appropriate concentrations with methanol. The diluted solutions were injected into UPLC for analysis. The LODs for GA, EA, hyperoside, astragalin, and kaempferol-3-*O*-sophoroside were 0.04, 0.08, 0.5, 0.2, and 0.08 µg/mL, respectively, which indicated that the analytical method was acceptable with sufficient sensitivity.

The recovery test was determined with a standard addition method. The samples (Sample 1) were spiked with high, intermediate, and low levels of mixed standard solutions of five components in triplicate, then extracted, processed, and quantified in accordance with established procedures. The results of the recovery rates are summarized in Table 1. The recovery rates were performed using a Waters Acquity BEH Shield C18 column (100 mm × 2.1 mm i.d., 1.8 µm). The recovery rates of the five compounds were in the range of 94.2%–103.8%, and their RSD values were less than 2.1%. Therefore, the UPLC-DAD methods were precise, accurate, and sensitive enough for the simultaneous quantitative evaluation of the five compounds in *R. rugosa*.

### 2.4. Method Validation of UPLC Fingerprint Analysis

The method of UPLC fingerprint analysis was validated with precision, repeatability, and stability tests. Intraday precision and repeatability as well as inter-day stability of the UPLC fingerprint method were determined and expressed by the relative standard deviations (RSD) value of the average relative retention times (RRT) and relative peak areas (RPA) of the 23 characteristic common peaks, with a reference peak (Peak 8) at retention time (t_R_) of 30.7 min. By using the optimized conditions described above, the repeatability of the UPLC method was calculated by analysis of five independently prepared solutions of the same sample (Sample 8). The variation in the RRT and RPA of the characteristic peaks did not exceed 2.3% and 2.5%, respectively. The intra-day precision variation of the RRT and RPA of the characteristic peaks was below 1% and 3%, respectively. This was obtained by the continuous intra-day analysis of the five replicate sample solutions (Sample 8). The inter-day stability test was assessed via analysis of the same sample solution (Sample 8) on two consecutive days at different time intervals (0, 6, 12, 24, and 48 h), and the RSD values of RRT as well as RPA of the characteristic common peaks were less than 2.3% and 2.5%, respectively. The observed results indicated that the sample solution was stable within 48 h. The results of the precision, stability, and repeatability tests are shown in Table 2, which met the national standards of traditional Chinese medicine fingerprinting (SFDA, 2000) [13].

**Figure 3:**
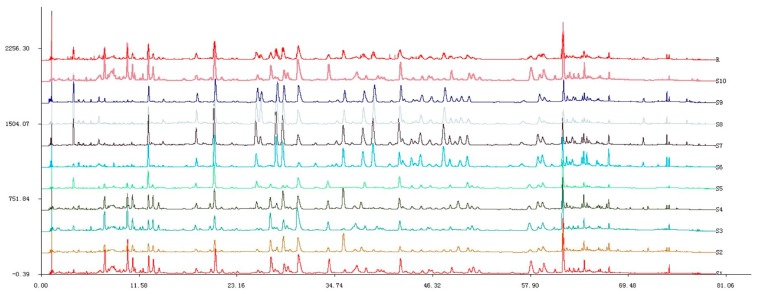
UPLC chromatographic fingerprints of 10 *R. rugosa* samples.

**Figure 4:**
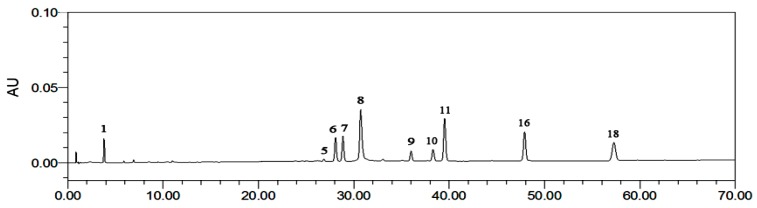
The reference fingerprint of *R. rugosa*: (1) GA (3.783 min); (5) 2-phenylethyl-*O*-β-d-glucopyranoside (26.9 min); (6) quercetin-3-O-(2′′-*O*-β-d-glucopyranosyl)-β-d-glucopyranoside) (28.0 min); (7) juglanin (28.9 min) (8) EC (30.7 min); (9) avicularin (36.0 min); (10) quercetin (38.3 min); (11) kaempferol-3*-O*-sophoroside (38.9 min) (16) hyperoside (48.9 min); (18) astragalin (57.3 min).

To standardize the fingerprint, 10 samples were analyzed. Software called Similarity Evaluation System for Chromatographic Fingerprint of Traditional Chinese Medicine (Version 2004A) [14] (Figure 3) was used to evaluate chromatograms. These samples had similar UPLC profiles. Peaks that existed in all 10 samples with relatively high intensity and good resolutions were assigned as “characteristic common peaks” for identification of the plant. There were 23 characteristic peaks (from Peaks 1 to 23) found in the chromatogram, which covered more than 65% of the total area. Ten components were identified by comparing their retention time and UV spectrum with those of standard compounds: Peak 1: GA; Peak 5: 2-phenylethyl-*O*-β-d-glucopyranoside; Peak 6: quercetin-3-*O*-(2′′-*O*-β-d-glucopyranosyl)-β-d-glucopyranoside); Peak 7: juglanin (28.9 min); Peak 8: EC (30.7 min); Peak 9: avicularin (36.0 min); Peak 10: quercetin (38.3 min); Peak 11: kaempferol-3-*O*-sophoroside (38.9 min); Peak 16: hyperoside (48.9 min); Peak 18: astragalin (57.3 min) (Figure 4). The other 13 common fingerprint peaks were unknown. To calculate the RRT and RPA of each characteristic peak, a reference peak should be chosen. Ellagic acid (Peak 8) had a considerably high content of more than 0.46% of the total area, and it also had moderate retention time, a stable peak area, and a good shape in the *R. rugosa* chromatograms. Therefore, it was chosen as the reference peak. Then, the retention time and peak area of the 23 common peaks were measured; RRT and RPA of all characteristic common peaks with respect to this reference peak were calculated (Table 3).

### 2.5. Similarity Analysis of HPLC Fingerprints of R. rugosa Samples

The similarities between the entire chromatographic profiles of 10 batches of *R. rugosa* and the standard chromatographic fingerprint were calculated with Similarity Evaluation System for Chromatographic Fingerprint of Traditional Chinese Medicine (Version 2004A), and the correlation coefficients of all 10 sample fingerprints were shown to be 0.991, 0.995, 0.994, 0.998, 0.999, 0.981, 0.997, 0.998, 0.998, and 0.999. These results showed that the 10 batches of *R. rugosa* from different plantations shared nearly the same correlation coefficients of similarities. In general, the common pattern of the 10 batches of test samples could be applied as a reference UPLC fingerprint to identify and assess *R. rugosa*.

### 2.6. Quantitative Determination of Ten Components in R. rugosa

In this study, the proposed UPLC method was successfully applied to the simultaneous determination of GA, EA, kaempferol-3-*O*-sophoroside, hyperoside, and astragalin in *R. rugosa* samples. The identity of the marker compound peaks in the chromatogram was confirmed by their retention times and their UV profiles. Quantification was based on the external standard method using calibration curves fitted by linear regression analysis. The contents of the five marker compounds in ten batches of the external standard method from different areas of *Rosa rugosa* are summarized in Table 4.

Contents of the hyperoside 92.8, astragalin 0.5, and GA 0.4 (mg/g) were significant, and EA yielded the lowest amount 0.4 (mg/g) in this study, while the amount of kaempferol-3-*O*-sophoroside was 0.4 (mg/g). The %RSD are the results of the three replicate injections of the plant extracts. From our literature review regarding the plant species under study, it appears that these five compounds have not been quantified before and are reported for the first time in this paper.

Samples of S1–S5 of *R. rugosa* were collected from northern Xinjiang, while the last parts were collected from southern Xinjiang. The average contents of hyperoside, astragalin, and kaempferol-3-*O*-sophoroside and GA in *R. rugosa* collected from the southern Xinjiang were higher than those from the northern Xinjiang. The average contents of EA from the north were higher than those from the south (Table 4).

## 3. Materials and Methods

### 3.1. Materials

Ten batches of raw material samples of *R. rugosa* were collected from Xinjiang Uyghur Autonomous Region, China. S1 was collected from Qitai of Xinjiang. S2–S5 were collected from Jimsar of Xinjiang. S6 was collected from ShaChe of Xinjiang. S7 was collected from YuTan of Xinjiang. S8 was collected from HoTan of Xinjiang. S9 was collected from the Hotan county of Xinjiang. S10 was collected from MoYu of Xinjiang. All the voucher specimens identified by research fellow Guanmian Shen, Xinjiang Institute of Ecology and Geography, Chinese Academy of Sciences.

### 3.2. Reagents

Acetonitrile (Fisher, optima^®^, LC-MS grade, Fair Lawn, NJ, USA) and formic acid (Merck, EMSURE^®^, analytical grade, Darmstadt, Germany) were used. Water used in the experiment was deionized and further purified by the Milli-Q Plus water purification system (Millipore Ltd., Bedford, MA, USA). Other reagents and chemicals were of analytical grade. 

Standard preparation: The five chemical standards (hyperoside, gallic acid, ellagic acid, astragalin, and kaempferol-3-*O*-sophoroside were confirmed via UV and ESI-MS, and the chromatogram of the mixture standards is shown in Figure 1. The purity of each compound was determined to be higher than 98% by the normalization of the peak area detected via HPLC. The reference compounds were accurately weighed, dissolved in methanol, and diluted to appropriate concentration ranges for the establishment of the calibration curve. All stock and working standard solutions were stored at 4 °C until used for analysis.

Sample preparation: Dried and finely powdered flowers of *R. rugosa* were extracted with 60% aqueous ethanol (100 mL) for 1 h under ultrasonic. The solution was filtered through a 0.22 µm filter before UPLC analysis. 

### 3.3. UPLC Condition

UPLC analysis was performed on a Waters Acquity UPLC™ system (Waters, Milford, MA, USA) equipped with binary solvent delivery pump, an auto sampler, and a photodiode array detector (PAD). The instrument was controlled by Waters Empower 2 software. The chromatographic separation was performed using a Waters Acquity BEH Shield C18 column (100 mm 2.1 mm i.d., 1.8 µm, Waters), operated at 35 °C. The mobile phase consisted of 0.1% formic acid–acetonitrile (A) and 0.1% formic acid–water (B) with a gradient elution of 2% A (0–1 min), 2%–5% A (1–2 min), 5%–10% A (2–7 min), 10%–11% A (7–10 min), 11%–18% A (10–56 min), 18%–28% A (56–69 min), 28%–43% A (69–71 min). Chromatograms were recorded at an absorbance of 260 nm. The mobile phase was eluted at a flow rate of 250 µL·min^−1^, and the injection volume was 2.00 μL.

The standard curves were obtained by plotting the peak area against the nominal concentration of each compound and were fitted to a linear function of type *y* = a*x* + b. In this equation, *y* and *x* represent peak area and nominal concentration in mg/L, respectively. The limit of detection (LOD) was estimated as the minimum concentration of the compounds needed to produce signals that were at least three times stronger than the noise signal (S/N). The accuracy tests were carried out by the known contents of the mixed standard solution into the known concentration of *R. rugosa* samples, and the assessment was done by analyzing the three different concentrations in triplicate. The percent recovery rates for the analyses were presented as the mean of the three results.

The precision of the UPLC fingerprint method was determined by analyses of the replicated extraction solution of the same sample five times within a day. The sample stability test was determined with one sample on two consecutive days. The repeatability was assessed by analyses of five independently prepared extraction solutions of *R. rugosa* samples. During this period, the solution was stored at room temperature [15].

To establish the representative chromatographic fingerprint, 10 batches of *R. rugosa* samples were analyzed under the established UPLC method. The obtained UPLC data from 10 batches of *R. rugosa* samples were exported from Waters Empower 2 software in AIA format and imported to the professional software named Similarity Evaluation System for Chromatographic Fingerprint of Traditional Chinese Medicine (Version 2004A).This system could reflect the similarity of the distribution ratio of the chemical composition accurately, as recommended by the SFDA.

## 4. Conclusions

In this study, the UPLC-DAD method proved to be simple, accurate, and reliable for the developed UPLC fingerprint and the determination of five bioactive compounds in *R. rugosa*. For the fingerprint analysis, 23 characteristic fingerprint peaks were applied to evaluate the similarities among 10 batches of *R. rugosa*, and they showed good similarities. For the quantitative determination, five components of the 10 batches of *R. rugosa* were successfully separated and determined. The UPLC fingerprint method was well validated by systematically comparing chromatograms of all samples from different regions. The method developed in this study will provide an important reference to establish the quality control method for other related traditional Chinese medicinal preparations.

## Figures and Tables

**Table 1:** Recovery rates of the five components in *R. rugosa*.

Component	Original (mg/g)	Added (mg/g)	Found (mg/g)	Recovery Rate (%)	RSD (%)
Gallic acid	0.200	0.050	0.249	98.0%	1.6
	0.200	0.100	0.299	99.0%	1.8
	0.200	0.200	0.402	101.0%	1.0
Ellagic acid	0.460	0.120	0.573	94.2%	1.3
	0.460	0.230	0.683	97.0%	2.0
	0.460	0.460	0.904	96.5%	2.1
Hyperoside	118.870	29.720	148.030	98.1%	1.3
	118.870	59.430	177.370	98.4%	0.9
	118.870	118.870	236.910	99.3%	1.5
Astragalin	0.340	0.080	0.423	103.8%	0.9
	0.340	0.170	0.507	98.2%	2.1
	0.340	0.340	0.671	97.4%	1.7
Kaempferol-3-*O*-sophoroside	0.120	0.030	0.149	96.7%	2.3
	0.120	0.060	0.178	96.7%	1.9
	0.120	0.120	0.234	95.0%	1.4

**Table 2:** Analytical results of precision, stability, and repeatability tests of 23 characteristic common peaks in *R. rugosa* samples (Sample 8) (*n* = 5).

Peak No.	RSD of RRT (%)			RSD of RPA (%)		
	Precision	Stability	Repeatability	Precision	Stability	Repeatability
1	0.11	0.14	0.18	0.16	0.09	0.12
2	0.24	0.19	0.17	0.21	0.13	0.17
3	0.22	0.08	0.04	0.11	0.15	0.21
4	0.13	0.09	0.15	0.18	0.18	0.27
5	0.17	0.2	0.17	0.19	0.21	0.19
6	0.12	0.29	0.2	0.11	0.22	0.21
7	0.09	0.25	0.19	0.19	0.16	0.14
8(S)	0	0	0	0	0	0
9	0.08	0.21	0.22	0.11	0.19	0.18
10	0.11	0.22	0.13	0.12	0.08	0.26
11	0.19	0.13	15	0.13	0.23	0.25
12	0.16	0.14	0.27	0.21	0.12	0.16
13	0.17	0.19	0.18	0.19	0.22	0.26
14	0.2	0.17	0.17	0.09	0.21	0.17
15	0.12	0.14	0.22	0.06	0.2	0.26
16	0.18	0.23	0.11	0.16	0.11	0.19
17	0.19	0.15	0.16	0.24	0.25	0.15
18	0.15	0.2	0.1	0.16	0.07	0.14
19	0.12	0.17	0.11	0.18	0.16	0.18
20	0.13	0.14	0.26	0.11	0.17	0.19
21	0.1	0.29	0.11	0.23	0.14	0.14
22	0.23	0.26	0.19	0.21	0.15	0.15
23	0.25	0.27	0.23	0.21	0.16	0.17

**Table 3:** The retention time (t_R_), the relative retention time (RRT), the peak area (PA), and the relative peak area (RPA) of 23 common peaks in *R. rugosa* (*n* = 10).

Component	t_R_ (min)	RRT		PA (mVs)	RPA	
		Average	RSD (%)		Average	RSD (%)
1	3.8	0.123	0.06	189,231	0.026	0.17
2	12.6	0.412	0.11	1,729,467	0.236	0.10
3	18.3	0.597	0.19	909,481	0.124	0.19
4	20.4	0.666	0.03	3,648,219	0.498	0.28
5	26.9	0.874	0.13	1,834,719	0.251	0.25
6	28.1	0.914	0.23	3,984,770	0.544	0.09
7	28.9	0.940	0.18	3,443,018	0.470	0.25
8	30.7	1.000	0.00	7,320,021	1.000	0.00
9	36.0	1.172	0.18	2,647,067	0.362	0.23
10	38.3	1.248	0.10	2,577,145	0.352	0.15
11	39.5	1.287	0.17	3,672,742	0.502	0.01
12	42.4	1.380	0.17	1,878,557	0.257	0.26
13	43.8	1.519	0.12	1,391,505	0.190	0.24
14	44.9	1.462	0.13	2,255,259	0.308	0.14
15	47.9	1.560	0.22	3,443,432	0.470	0.16
16	48.4	1.575	0.01	1,227,205	0.168	0.17
17	50.4	1.642	0.08	1,738,039	0.237	0.25
18	57.3	1.864	0.16	550,902	0.075	0.26
19	58.8	1.915	0.19	1,846,625	0.252	0.19
20	59.3	1.932	0.07	2,328,296	0.318	0.11
21	61.8	2.011	0.21	2,595,214	0.355	0.19
22	64.6	2.104	0.14	984,376	0.134	0.14
23	67.2	2.189	0.19	1,214,014	0.166	0.12

**Table 4:** Contents of 10 components in *R. rugosa* (*n* = 3, mg/g).

Sample No.	Content of Investigated Components				
	Gallic Acid	Ellagic Acid	Hyperoside	Astragalin	Kaempferol
S1	0.20	0.46	118.87	0.34	0.12
S2	0.17	0.43	10.03	0.31	0.11
S3	0.37	0.30	46.76	0.47	0.10
S4	0.19	0.42	75.65	0.29	0.09
S5	0.19	0.28	55.16	0.51	0.05
Average (S1–S5)	0.22	0.38	61.29	0.38	0.09
S6	0.43	0.32	82.76	0.53	0.05
S7	0.23	0.19	123.80	0.62	0.77
S8	0.79	0.49	203.40	1.12	1.20
S9	0.88	0.40	136.18	0.60	0.75
S10	0.37	0.31	75.65	0.44	0.48
Average (S6–S10)	0.54	0.34	124.36	0.66	0.65
Average (S1–S10)	0.38	0.36	92.83	0.52	0.37
RSD (%)	0.67	0.26	0.59	0.46	1.09
